# Calcium-Independent Gelation of *Abelia macrotera* Pectin Induced by Sodium Ions and Its Synergistic Interaction with Pea Protein Isolate

**DOI:** 10.3390/foods15101782

**Published:** 2026-05-18

**Authors:** Jianglin Wang, Wanting Li, Shunhong Hu, Binrong Sun, Xiankang Fan, Jie Luo, Qiqi Mao, Hui Zhou

**Affiliations:** College of Food Science and Technology, Hunan Agricultural University, Changsha 410128, China; 17363897816@139.com (J.W.);

**Keywords:** *Abelia macrotera* pectin, low-methoxyl pectin, sodium-induced gelation, pea protein isolate

## Abstract

The effects of Na^+^ and pea protein isolate (PPI) concentrations on the gelation behavior of *Abelia macrotera* pectin (AMP) were systematically investigated using texture analysis, Fourier-transform infrared spectroscopy (FTIR), and scanning electron microscopy (SEM). AMP formed stable gels in the presence of Na^+^ without requiring Ca^2+^, and gel properties strongly depended on Na^+^ concentration and a transition from dense to loose microstructures with increasing Na^+^ concentrations. Optimal gel performance was achieved at Na^+^ concentrations of 0.15–0.20 mol/L. The AMP–PPI composite gel exhibited the optimal performance at an AMP:PPI ratio of 0.3:7.5 and Na^+^ concentrations of 0.10–0.15 mol/L, showing enhanced textural properties, water-holding capacity, and network compactness. FTIR results revealed that Na^+^ induced non-covalent electrostatic and ion–dipole interactions without forming new covalent bonds. These findings provide a theoretical basis for developing sodium-regulated, calcium-free pectin–protein gels.

## 1. Introduction

Pectin is a natural polysaccharide found in plant cell walls and is widely used in the food, pharmaceutical, and cosmetic industries owing to its gelling properties. Gelling performance is influenced by several factors, such as ionic strength and plant protein isolate concentration [1]. Molecular weight and cross-linking degree regulate intermolecular hydrogen bonding, electrostatic interactions, and hydrophobic interactions, which collectively influence gel texture, water-holding capacity (WHC), and rheological behavior [2]. According to classical theory, high-methoxyl pectin (DM > 50%) requires 65% sugar and low pH (3.5) for gelation, whereas low-methoxyl pectin (LMP, DM < 50%) forms gels through divalent cations (e.g., Ca^2+^) using the “egg-box model” [3]. Additionally, LMP can form acid-induced gels by reducing electrostatic repulsion between protonated carboxyl groups, independent of divalent cations [4]. Recent studies have shown that pectin gelation can also occur with monovalent cations, such as Na^+^, through a mechanism that is fundamentally different from divalent cation-mediated crosslinking. Wang et al. [5] reported that Na^+^ facilitated high-ester pectin gelation by reducing charge density through electrostatic shielding following de-esterification under alkaline conditions. Additionally, Na^+^ induces gelation of sunflower pectin at a pH of 3.0–5.0 [6]. In Na^+^-induced gelation, monovalent ions mainly shield electrostatic repulsion between negatively charged carboxylate groups on polymer chains, resulting in relatively weak, reversible aggregation and formation of a soft, ionic-strength-dependent network.

Plant pectin is a natural polysaccharide that has a wide range of applications in food, medicine, and cosmetics [7]. *Abelia macrophylla* is a valuable medicinal plant native to China, and its leaves contain abundant pectin. Mao et al. [8] reported that pectin extracted from *Abelia macrotera* (AMP) exhibited low esterification (DE = 1.17%), high galacturonic acid content (approximately 72.16%), an RG-I type structure, and a molecular weight of 1392 Da, and the main monosaccharide components included Glc, Ara, Gal, Rha, Man, Xyl, and GlcN. Although Na^+^-induced gelation has been reported for some low-methoxyl pectins (LMPs) from other plant sources, nearly all reported Na^+^-responsive LMPs are homogalacturonan (HG)-dominant pectins with a moderate degree of esterification (DE, 20–50%), and their Na^+^-induced gelation strictly relies on specific harsh conditions: for example, citrus high-ester pectin requires alkaline de-esterification to generate free carboxyl groups before Na^+^-mediated gelation [9], while sunflower head pectin only forms Na^+^-induced gels within a narrow acidic pH range of 3.0–5.0 [10]. By contrast, the pectin extracted from *Abelia macrophylla* (AMP) possesses distinct structural features that set it apart from previously studied LMPs. These structural characteristics enable AMP to achieve stable Na^+^-induced gelation under mild conditions without alkaline pretreatment or strict pH limitation, which cannot be achieved by the conventional LMPs reported in previous studies.

In recent years, plant protein gels have attracted growing interest in the food industry as alternatives to animal-based meat and dairy products and for functional food applications owing to their nutritional and functional properties [11]. Pea protein isolate (PPI), a high-quality plant protein, has high nutritional value and good gel-forming ability but exhibits poor gel performance due to weak intermolecular interactions [12]. AMP, an anionic polysaccharide, improves gel network structure through electrostatic or hydrophobic interactions with proteins. The composite gelation of AMP and PPI is influenced by multiple factors. However, the regulatory role of ionic strength (particularly Na^+^ concentration) remains unexplored. Sodium ions (Na^+^), common ionic additives in food systems, enhance protein and polysaccharide solubility and alter intermolecular interactions through charge shielding effects, thereby modifying gel microstructure and macroscopic properties.

Currently, the synergistic regulatory mechanisms by which Na^+^ concentration influences the structural and functional properties of AMP–PPI composite gels remain unclear. Therefore, in this study, the effects of Na^+^ and PPI concentrations on AMP gel properties were systematically investigated. Additionally, the AMP–PPI composite system was used to evaluate the effect of Na^+^ concentration (0–0.25 mol/L) on gel apparent morphology, texture characteristics, WHC, rheological behavior, and microstructure. Multi-scale characterization clarified the Na^+^-dependent gelation mechanism and established a “structure–property” relationship, providing a theoretical basis for AMP applications in food systems.

## 2. Materials and Methods

### 2.1. Materials

Analytical-grade sodium chloride (NaCl), hydrochloric acid (HCl), and sodium hydroxide (NaOH) were purchased from China National Pharmaceutical Group Chemical Reagent Co., Ltd. (Beijing, China). Food-grade pea protein was sourced from Henan Zhongchen Biotechnology Co., Ltd. (Zhengzhou, Henan, China). The fresh, tender leaves of *Abelia macrotera* were collected from Jinshiqiao Town, Longhui County, Hunan Province, China.

Then, AMP was extracted from *Abelia macrotera* leaves using a modified method reported by Liu et al. [13]. Fresh leaves were washed with distilled water and dried in a blast drying oven at 60 °C for 24 h. The dried leaves were ground and sieved through a 60-mesh screen. Precisely 8 g of leaf powder was dispersed in 0.048 M ammonium oxalate solution at a solid-to-liquid ratio of 1:60 (*w*/*v*). The suspension was ultrasonically treated at 600 W for 20 min, stirred using a thermostatic magnetic stirrer (DF-101S, Zhengzhou Yarong Instrument Co., Ltd., Zhengzhou, China), and centrifuged (TG16-WS, Xiangyi Centrifuge Instrument Co., Ltd., Changsha, China) at 783× *g* for 10 min. The supernatant was collected through suction filtration. The residue was enzymatically hydrolyzed by adding cellulase (0.4% *w*/*w*, EC 3.2.1.4, 300 µkat/g), followed by the addition of fresh 0.048 M ammonium oxalate at a solid-to-liquid ratio of 1:30 (*w*/*v*). Hydrolysis was performed at 60 °C for 40 min, and enzymes were inactivated in a boiling water bath for 5 min. The hydrolysate was centrifuged (783× *g*, 5 min) and filtered. The ultrasonic and enzymatic supernatants were mixed. Four volumes of anhydrous ethanol were added, and the mixture was thoroughly stirred and incubated at 4 °C for 12 h. The precipitate was collected through suction filtration, repeatedly washed with ethanol until decolorized, and redissolved in 500 mL of distilled water. The solution was dialyzed for 48 h and freeze-dried using a vacuum freeze dryer (LGJ-25E, Four Rings Furui Instrument Technology Development Co., Ltd., Beijing, China) for 72 h.

#### 2.1.1. Preparation of AMP Gel

AMP gels were prepared using a modified version of Zhao et al.’s method [2]. A defined amount of AMP powder was dissolved in deionized water and magnetically stirred at ambient temperature (25 °C) for 1 h. The pH of the AMP solution was adjusted to 4.5 ± 0.05 using 0.1 mol/L HCl. Then, a predetermined concentration of NaCl solution was slowly added under continuous stirring (68× *g*). The mixture was incubated at 4 °C for 12 h, and the gel strength, WHC, and rheological properties of the resulting gels were analyzed.

#### 2.1.2. AMP–PPI Gel Preparation

PPI powder was dissolved in deionized water, magnetically stirred at room temperature for 12 h, and allowed to equilibrate at room temperature before use. AMP–PPI gels were prepared using a modified method reported by Pan et al. [9]. At 25 °C, AMP solution (1 g/hg) and PPI solution (25 g/hg) were mixed at specified ratios, and ultrapure water was added to adjust final concentrations to AMP (0, 0.1, 0.2, 0.3, 0.4, and 0.5 g/hg) and PPI (0, 2.5, 5, 7.5, 10, and 12.5 g/hg). The pH was adjusted to 6.5 using 0.1 mol/L HCl, and the mixture was stirred at 68× *g* for 1 min. Samples were heated in a 95 °C water bath under continuous magnetic stirring for 10 min. Afterward, the samples were refrigerated at 4 °C for 12 h to induce gelation.

### 2.2. Methods

#### 2.2.1. Gel Hardness Measurement

Gel hardness of AMP and AMP–PPI gels was measured using texture profile analysis with a modified method reported by Lu et al. [14]. The test parameters were as follows: pre-test speed of 2 mm/s, trigger force of 5 g, test speed of 1 mm/s, post-test speed of 2 mm/s, compression deformation of 50%, and interval time of 5 s. Measurements were conducted at room temperature (25 °C).

#### 2.2.2. WHC Measurement

WHC was evaluated using a modified method of Zhang et al. [15]. Aliquots (5 g) of AMP and AMP–PPI gels were centrifuged at 1, 118× *g* for 15 min. The supernatant was discarded, and tubes were inverted for 5 min to drain excess water. WHC was calculated as the ratio of gel mass after centrifugation to initial gel mass, using the following formula:

$$\mathrm{WHC}\;=\;\frac{W_{1}-W_{0}}{W_{2}-W_{0}}\times 100\%$$

where W_0_ is the mass of the empty centrifuge tube (g), W_1_ is the mass of tube + gel after centrifugation (g), and W_2_ represents the mass of tube + initial gel sample (g).

#### 2.2.3. Rheological Measurement

The viscoelastic properties of the gel samples were characterized using a rotational rheometer (Kinexus Pro^+^, Malvern Instruments Ltd., Malvern, UK). Measurements were conducted following the modified method reported by Cao et al. [16]. Before frequency sweep tests were conducted, strain amplitude sweep experiments (0.01–10%) were performed at 25 °C to determine the linear viscoelastic region, and a strain of 1% was selected. A parallel plate geometry (PP, 20 mm diameter) was used with a fixed gap of 1.0 mm. After loading, the samples were allowed to equilibrate for 5 min at the test temperature. Temperature was controlled at 25 °C using a Peltier temperature control system with an accuracy of ±0.1 °C. Frequency sweep was measured within a range of 0.1–10 Hz, and the storage modulus (G′) and loss modulus (G″) were recorded.

#### 2.2.4. Effect of Na^+^ Concentration on AMP Gel

At 25 °C, a series of mixtures was prepared with NaCl solutions (0.1, 0.15, 0.20, 0.25, 0.30, and 0.35 mol/L) and 1 g/hg pectin solution (pH adjusted to 4.5 using 0.1 mol/L HCl). Each sample was adjusted to a final volume of 5 mL. Then, the prepared mixtures were allowed to stand at 4 °C for 12 h to observe gelation behavior.

#### 2.2.5. Fourier-Transform Infrared Spectroscopy Analysis of AMP–PPI Gels

A specified amount of dry potassium bromide (KBr) powder was added to pectin powder in a mortar with a pestle and thoroughly ground to ensure homogeneous mixing. The ground mixture was transferred to a pellet die and compressed under uniform pressure (1.5 Mpa) for 30 s to form a transparent or translucent thin film sample. A Fourier-transform infrared spectrometer (FTIR, IRAffinity-1, Shimadzu Corporation, Kyoto, Japan) was used to scan the sample in the range of 400–4000 cm^−1^, and the spectrum was recorded [17].

#### 2.2.6. Scanning Electron Microscope Observations of AMP–PPI Gels

An appropriate amount of dried gel was placed onto a conductive adhesive sample processing disk and sputter-coated with gold. The cross-sectional microstructure of the gel was examined using a scanning electron microscope (SEM, JSM-6380LV, JEOL Ltd., Tokyo, Japan), and photographs were taken. The acceleration voltage was 10 kV, with magnification factors of 50×, 200×, and 500×.

#### 2.2.7. Data Analysis

All analyses were performed in triplicate, and results are expressed as the mean ± standard deviation. Analysis of variance was performed on all data using SPSS software (IBM SPSS Statistics 28). When a statistically significant overall difference was detected (*p* < 0.05), Duncan’s multiple range test was subsequently used as a post hoc test to identify specific differences between groups. Graphs were generated using Origin 2024 software.

## 3. Results and Discussion

### 3.1. AMP Gel Properties

#### 3.1.1. Apparent Morphology of Na^+^-Induced AMP Gels

**Figure 1:**
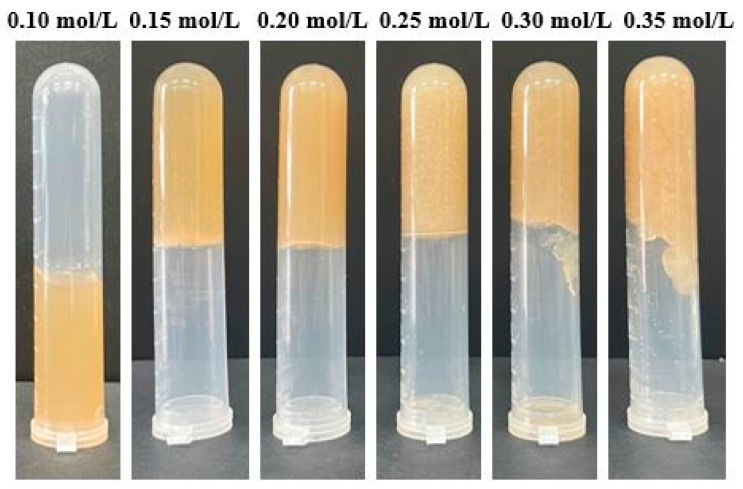
Apparent morphology of AMP gels with different Na^+^ concentrations.

Na^+^ acts primarily through electrostatic shielding and non-covalent (e.g., ion–dipole) interactions, reducing repulsion between negatively charged pectin chains [18]. The gelation behavior of AMP solutions was mainly influenced by Na^+^ concentration (Figure 1). At 0.10 mol/L Na^+^, the system remained fluid with only minor flocculation, indicating that electrostatic repulsion between pectin chains remained dominant due to insufficient charge shielding. At a Na^+^ concentration of 0.15–0.20 mol/L, transparent and homogeneous gels were formed. This formation indicated that an optimal level of electrostatic shielding was achieved, promoting pectin chain association and the formation of a coherent network. However, a further increase in Na^+^ concentration (0.25–0.35 mol/L) led to gel softening, inhomogeneity, and syneresis. This transition aligns with a salting-out mechanism, where excessive ions compete for water molecules and disrupt the hydration shells around the pectin chains, thereby compromising the gel network integrity [11,19].

#### 3.1.2. Textural Properties

The textural parameters of AMP gels non-monotonically varied with Na^+^ concentration (Table 1). The hardness, elasticity, and chewiness of the gel reached optimal values at 0.30 mol/L Na^+^ before declining at 0.35 mol/L. This phenomenon was attributed to the dual role of Na^+^: at moderate concentrations (up to 0.30 mol/L), Na^+^ screened electrostatic repulsion between pectin chains, enabling denser network formation, and improved mechanical strength and elasticity. The simultaneous increase in chewiness and cohesiveness further confirms the development of a strong, integrated structure. However, at 0.35 mol/L, the onset of a salting-out effect disrupted this balance. Excessive ions compete for water, partially dehydrating and collapsing the network, leading to the observed decrease in all textural parameters [20,21]. This deterioration in macroscopic texture was consistent with the observed syneresis and loss of homogeneity at high Na^+^ concentrations (Figure 1).

#### 3.1.3. WHC Measurement

**Figure 2:**
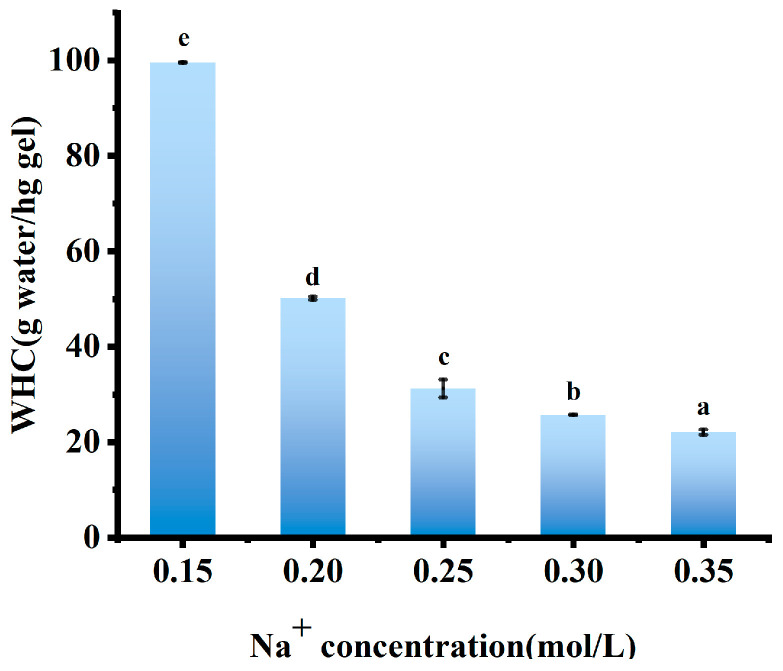
WHC of AMP with different Na^+^ concentrations. Note: Different lowercase letters indicate significant differences between groups (*p* < 0.05).

WHC of AMP gels monotonically decreased as Na^+^ concentration increased from 0.15 to 0.35 mol/L (Figure 2). This trend reflected the changing role of Na^+^ from a network promoter to a network disruptor. At the optimal gelation concentration (0.15–0.20 mol/L, Figure 1), Na^+^ provided sufficient electrostatic screening to allow network formation without compromising hydration, thereby leading to high WHC [22]. However, as the concentration of Na^+^ increased, the same screening effect that initially promoted gelation began to reduce WHC. Enhanced charge shielding facilitated closer chain association and aggregation, which reduced the number of available water-binding sites and compressed the network pores, partially expelling water [9]. Therefore, at 0.30 mol/L, the gel exhibited the highest hardness (Table 1) but had a lower WHC, indicating that the network was compact but less hydrophilic. At 0.35 mol/L, the salting-out effect became dominant. Excess ions severely compete for water molecules, stripping the hydration shells from the pectin chains and causing catastrophic dehydration and network collapse. This phenomenon was confirmed by the concurrent rapid decrease in WHC, hardness, and elasticity [9,19].

#### 3.1.4. Rheological Measurements

**Figure 3:**
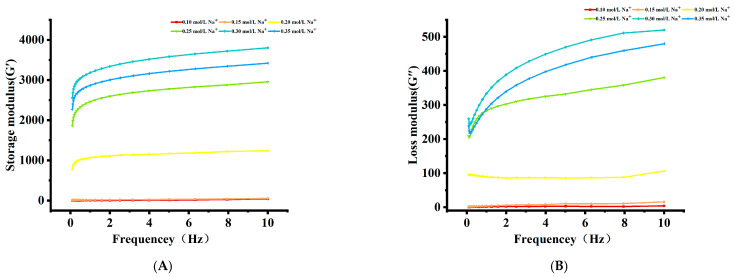
Effect of AMP with different Na^+^ concentrations on the (**A**) storage modulus (G′) and (**B**) loss modulus (G″).

The viscoelasticity of AMP gels, characterized by the storage modulus (G′) and loss modulus (G″), was significantly influenced by Na^+^ concentration (Figure 3A,B). At 0.10 mol/L Na^+^, G″ predominated over G′, confirming a viscous fluid state where electrostatic repulsion prevented permanent network formation [23]. When Na^+^ concentration exceeded 0.15 mol/L, G′ surpassed G″ across the measured frequency range, indicating the establishment of a true, elastic gel network [6]. Both moduli increased with frequency, a typical behavior of soft solids. Notably, at any given frequency, G′ and G″ displayed a non-monotonic dependence on Na^+^ concentration, both peaking at 0.30 mol/L. This optimum correlated with the maximum gel hardness (Table 1) and reflected the apex of Na^+^-mediated network strengthening. The optimal electrostatic screening facilitated extensive inter-chain associations, forming a strong network that efficiently stores (high G′) and dissipates (high G″) mechanical energy [24]. The subsequent decline in both moduli at 0.35 mol/L Na^+^ was consistent with the salting-out regime, where excessive ions disrupted the hydration and integrity of the gel network.

### 3.2. Characteristics of AMP–PPI Complex Gel

#### 3.2.1. Apparent Morphology

**Figure 4:**
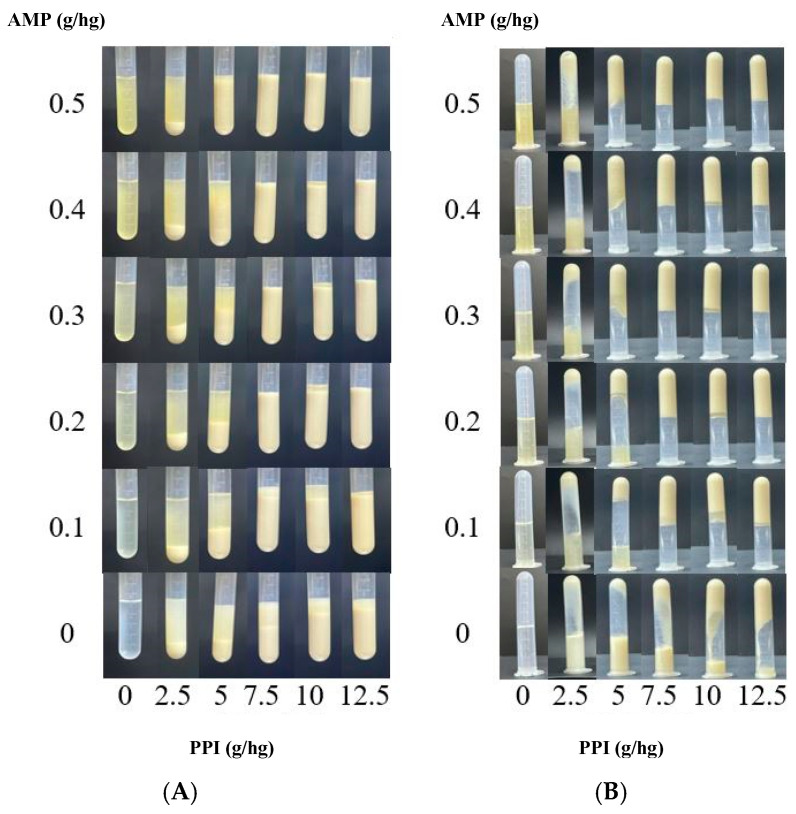
Photographs of AMP–PPI mixtures in (**A**) upright and (**B**) inverted positions.

The phase behavior and gelation of AMP–PPI mixtures at varying ratios are summarized in Figure 4. At the experimental pH (6.5), both AMP and PPI carried net negative charges, leading to thermodynamic incompatibility and phase separation at low biopolymer concentrations, as expected for such electrostatically repulsive systems [25]. However, when the concentration exceeded specific thresholds, homogeneous composite gels were formed. This transition indicates that AMP can effectively promote network formation in PPI, acting as an anionic polysaccharide crosslinker [26]. The texture and WHC of these stable gels were measured (Table 2). The formulation with 0.3 g/hg AMP and 7.5 g/hg PPI exhibited the highest hardness and WHC, representing an optimal balance between cross-linking density and network integrity. Excessive cross-linking, as observed at higher AMP ratios, can conversely impair textural properties [27]. Therefore, the AMP:PPI ratio of 0.3:7.5 was selected for all subsequent investigations into the effects of Na^+^ concentration.

#### 3.2.2. Apparent Morphology of Na^+^-Induced AMP–PPI Composite Gels

**Figure 5:**
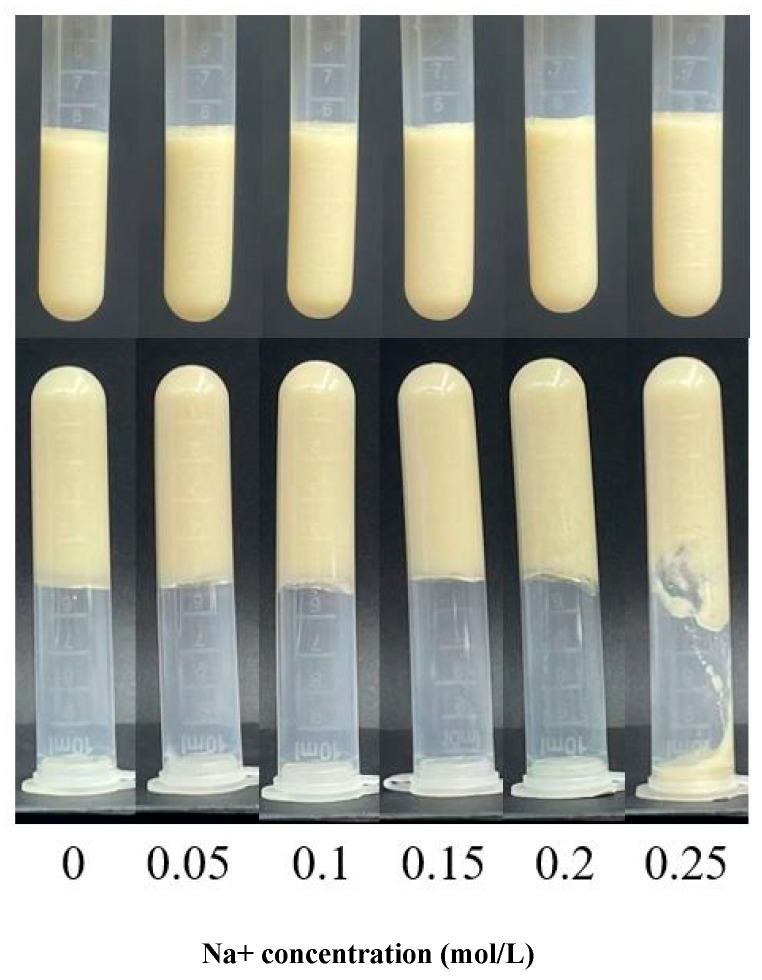
Visual appearance of AMP–PPI composite gels (upright and inverted) with different Na^+^ concentrations.

The macroscopic structure of the optimal AMP–PPI composite gel was mainly influenced by Na^+^ concentration (Figure 5). In the absence of Na^+^, slight syneresis was observed, indicating a weak network where intermolecular electrostatic repulsion limited effective cross-linking. The introduction of Na^+^ at low concentrations (0.05–0.15 mol/L) eliminated syneresis and yielded dense, uniform gels. Under these conditions, Na^+^ ions acted to screen the negative charges on both biopolymers, reducing electrostatic repulsion and promoting intermolecular association and the development of a cohesive composite network [28,29]. However, beyond an optimal point (≥0.20 mol/L), Na^+^ became detrimental, inducing severe syneresis and structural collapse. This breakdown aligns with a salting-out mechanism, where excessive ions disrupt the hydration shells critical for network stability, resulting in excessive aggregation and phase separation [30,31].

#### 3.2.3. Textural Properties

The textural characteristics of AMP–PPI gels at various Na^+^ concentrations are summarized in Table 3. Textural parameters (including hardness, elasticity, adhesiveness, chewiness, and cohesiveness) initially increased then decreased with increasing Na^+^ concentration (0–0.20 mol/L), and optimal performance was observed at 0.15 mol/L Na^+^. The gel system exhibits optimal stability when the Na^+^ concentration in AMP gels is 0.2 mol/L. The likely reason is that the presence of PPI in the composite gel system increases the total negative charge density and makes it more sensitive to salts, resulting in a lower optimal Na^+^ concentration. At lower concentrations, Na^+^ reduced electrostatic repulsion between AMP and PPI molecules, enhancing intermolecular interactions and promoting gel network formation and consolidation. As a result, gel became harder, more elastic, more adhesive, and chewier. Positively charged sodium ions interacted with negatively charged groups on pectin and protein molecules, causing aggregation and compaction of the three-dimensional network structure [31]. At 0.20 mol/L Na^+^, increased ionic strength thickened the hydration layer around AMP and PPI, enhancing electrostatic repulsion between molecules and disrupting the gel network. As a result, hardness and elasticity decreased, along with other textural properties, reducing gel network density and intermolecular interaction strength [32].

#### 3.2.4. WHC Measurement

**Figure 6:**
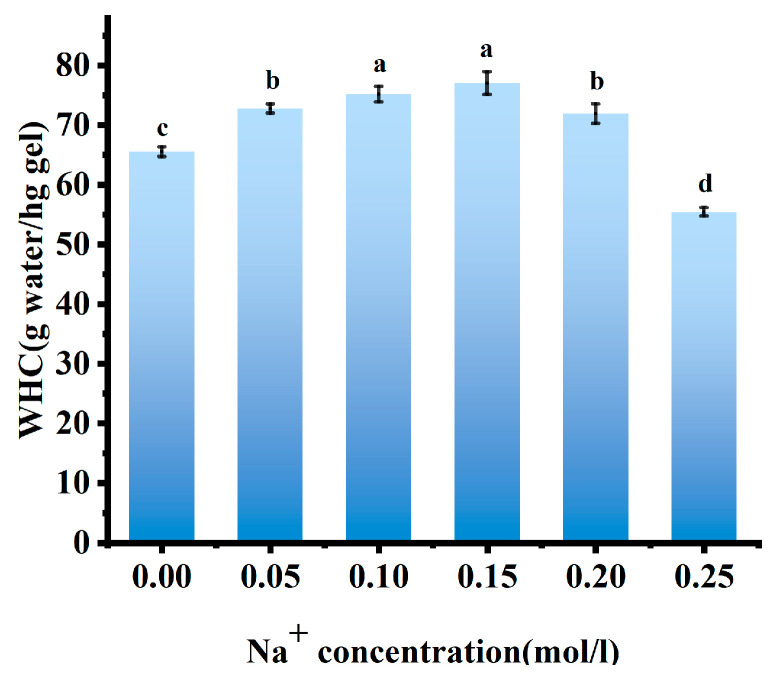
WHC of AMP–PPI composite gels with different Na^+^ concentrations. Note: Different lowercase letters indicate significant differences between groups (*p* < 0.05).

The optimal WHC of the AMP–PPI composite gel was achieved at 0.15 mol/L Na^+^ before declining at higher concentrations (Figure 6). This non-monotonic trend mirrors the evolution of gel network integrity. At the optimal Na^+^ concentration, effective charge screening reduced inter-biomolecule repulsion, facilitating the formation of a finely structured, cohesive network capable of immobilizing water through capillary forces and enhanced hydration [33]. Beyond this point, excessive Na^+^ ions trigger a salting-out effect. This phenomenon disrupted the hydration shells around the polymers, facilitated excessive hydrophobic aggregation, and ultimately compromised the gel matrix, leading to syneresis and a significant decrease in WHC [28].

#### 3.2.5. Rheological Properties

**Figure 7:**
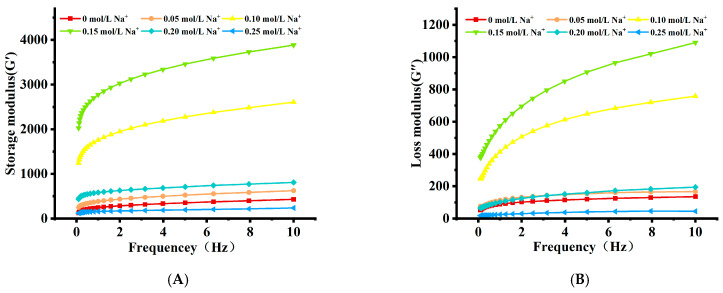
Effect of AMP–PPI composite gels with different Na^+^ concentrations on the (**A**) storage modulus (G′) and (**B**) loss modulus (G″).

The dynamic rheological properties of the AMP–PPI composite gel revealed its solid-like behavior and a pronounced dependence on Na^+^ concentration (Figure 7A). Across all samples, the storage modulus (G′) consistently exceeded the loss modulus (G″) throughout the frequency sweep, confirming the formation of a stable, elastic three-dimensional network [34]. Notably, both G′ and G″ exhibited a clear optimum at 0.15 mol/L Na^+^ (Figure 7B). This peak in viscoelastic strength directly corresponded to the optimal network structure observed macroscopically (Figure 5) and in texture analysis (Table 3). At the optimal concentration, Na^+^ ions provided effective electrostatic screening, enabling maximal intermolecular association and the formation of a dense, cohesive network that optimally resisted deformation (high G′) and dissipated energy (high G″) [35]. Conversely, at higher Na^+^ concentrations (≥0.20 mol/L), decreases in both moduli indicated network degradation, consistent with a salting-out effect that disrupts interactions and compromises gel integrity [36].

#### 3.2.6. FTIR Analysis

**Figure 8:**
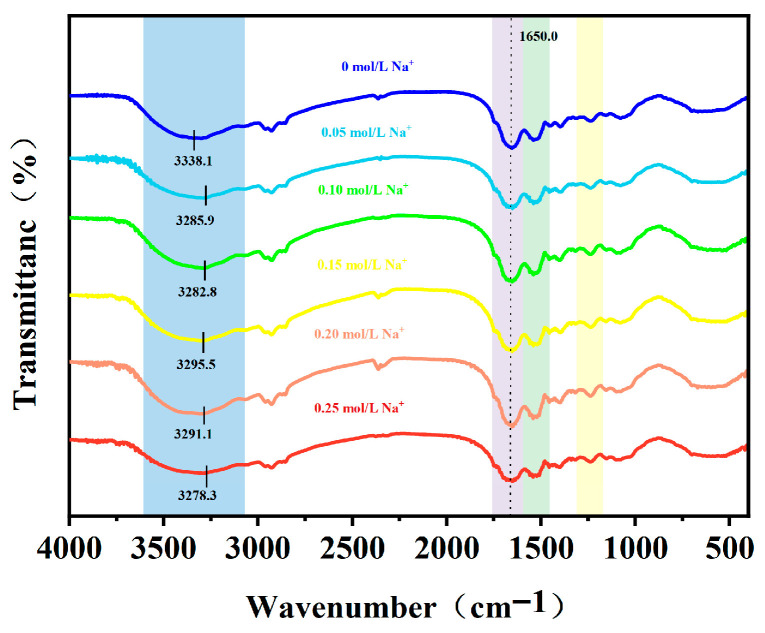
Fourier infrared spectrum of AMP–PPI composite gels with different Na^+^ concentrations.

FTIR analysis uncovered molecular interactions in the AMP–PPI composite gel (Figure 8). No new absorption peaks appeared in any sample across all Na^+^ gradients, confirming that Na^+^ mediates gel network formation solely through non-covalent interactions including electrostatic shielding, ion–dipole interactions and hydrogen bonding, without forming new covalent bonds or altering AMP and PPI’s primary covalent structure. Key spectral regions showed significant changes: the broad 3000–3600 cm^−1^ absorption band (assigned to O-H stretching of AMP pectin hydroxyl groups and N-H stretching of PPI peptide bond amide A band) redshifted continuously from 3338.1 cm^−1^ (0 mol/L Na^+^) to a minimum of 3282.8 cm^−1^ at 0.15 mol/L Na^+^, then blueshifted at higher concentrations, indicating a denser intermolecular hydrogen bond network formed in the gel [37]. Notably, the optimal hydration state was achieved at 0.15 mol/L Na^+^. The extremely weak absorption at 1720–1750 cm^−1^ (corresponding to pectin’s esterified carbonyl C=O stretching vibration) matched AMP’s ultra-low esterification degree (DE = 1.17%), and no significant peak shift occurred with increasing Na^+^ concentration, verifying Na^+^ has no effect on AMP’s esterification structure. The shoulder peak at 1600–1630 cm^−1^ (assigned to asymmetric stretching vibration of AMP’s free carboxylate groups -COO^−^, the core site for Na^+^-pectin interaction) reached maximum absorption intensity at 0.15 mol/L Na^+^, directly proving positively charged Na^+^ binds to negatively charged -COO^−^ via electrostatic and ion–dipole interactions, effectively shielding electrostatic repulsion between pectin chains. Meanwhile, in the amide I region (1650 cm^−1^), spectra evolved from a broad, gentle shape at 0 mol/L Na^+^ (indicating disordered protein structure and weak intermolecular interactions) to a sharper, intensified peak at 0.15 mol/L Na^+^, suggesting enhanced protein structural order and possible coordination between Na^+^ and carbonyl groups [38,39]. The absorption peaks in AMP’s 900–1200 cm^−1^ polysaccharide characteristic fingerprint region (corresponding to C-O-C glycosidic bond stretching and sugar ring skeleton vibrations) showed no significant shift across all samples, confirming Na^+^ does not alter AMP’s polysaccharide backbone. Collectively, these spectral changes verify that 0.15 mol/L Na^+^ is optimal for promoting intermolecular interactions between AMP and PPI and constructing a compact, stable gel network—consistent with the composite gels’ observed macroscopic texture, water-holding capacity, rheological properties and micromorphology. At excessive Na^+^ concentrations (≥0.20 mol/L), altered spectral features indicate disrupted hydration shells and weakened intermolecular interactions, aligning with the onset of salting-out effects.

#### 3.2.7. SEM Observations

**Figure 9:**
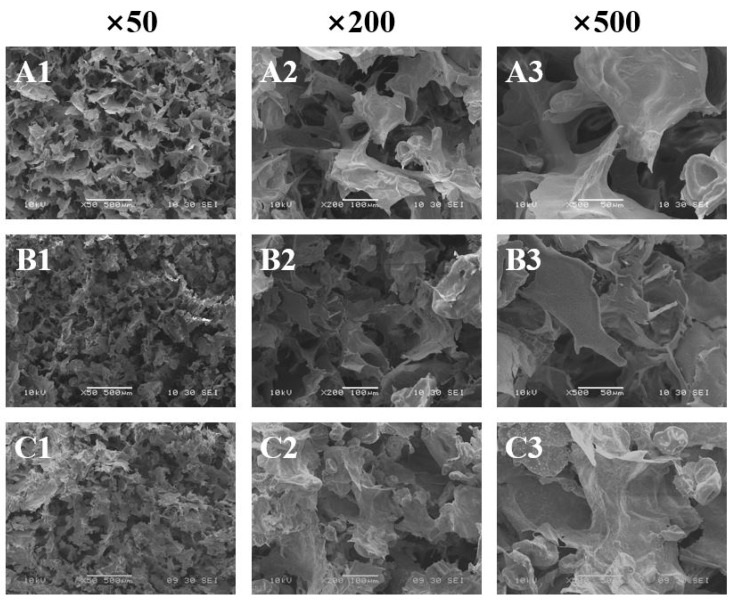

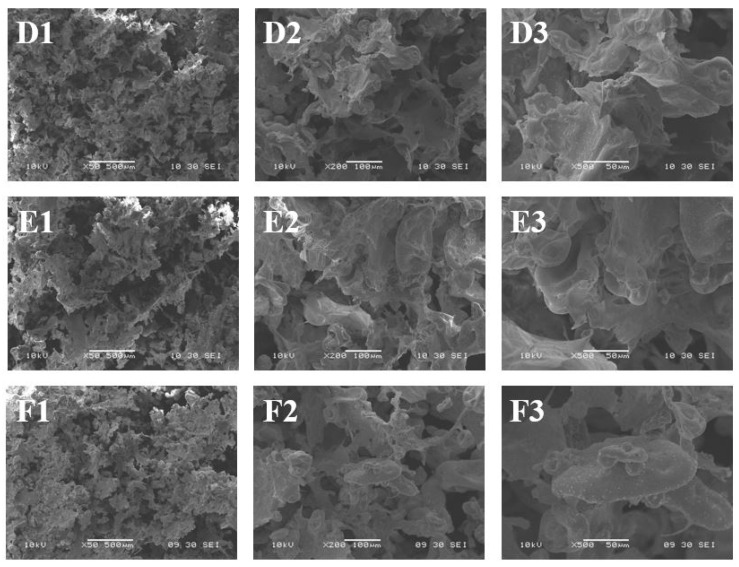
SEM images of AMP–PPI composite gels with different Na^+^ conditions: (**A**) 0 mol·L^−1^; (**B**) 0.05 mol·L^−1^; (**C**) 0.10 mol·L^−1^; (**D**) 0.15 mol·L^−1^; (**E**) 0.20 mol·L^−1^; and (**F**) 0.25 mol·L^−1^.

SEM directly revealed the evolution of the three-dimensional network microstructure in AMP–PPI composite gels as a function of Na^+^ concentration (Figure 9). The observed morphological transition—from a discontinuous, porous structure at 0 mol/L Na^+^ to a uniform, dense honeycomb-like network at 0.10–0.15 mol/L Na^+^, and finally to a collapsed, coarse structure at ≥0.20 mol/L Na^+^—provided direct visual evidence for the “dense-to-loose” trend inferred from macro- and molecular-scale analyses. This microstructural progression is influenced by the role of Na^+^. At optimal concentrations, Na^+^ reduced electrostatic repulsion and facilitated inter-biomolecular association, resulting in network consolidation and pore refinement [36]. Notably, in contrast to divalent cations (e.g., Ca^2+^), monovalent Na^+^ does not form specific ionic “egg-box” cross-links; instead, it mediates association mainly through non-specific electrostatic shielding and salting-in effects [40,41]. Conversely, at excessive concentrations, Na^+^ induced a salting-out effect, which promoted excessive aggregation and phase separation, thereby fragmenting the continuous network, as evidenced by the structural disintegration (Figure 9E,F) [38].

## 4. Conclusions

In this study, a sodium-dependent gelation mechanism of AMP and its synergistic interaction with PPI were elucidated. Na^+^ mainly reduced the electrostatic repulsion between negatively charged carboxyl groups on AMP chains, thereby promoting molecular aggregation and gel network formation. PPI further reinforced the network through hydrogen bonding and hydrophobic interactions with AMP, thereby enhancing its elasticity, hardness, and WHC. The AMP–Na^+^–PPI system formed a cooperative structure in which ionic coordination and non-covalent interactions collectively enhanced gel stability and compactness. At optimal Na^+^ concentrations (0.10–0.15 mol/L) and an AMP:PPI ratio of 0.3:7.5, the composite gel exhibited enhanced structural uniformity and superior viscoelastic performance. However, higher Na^+^ concentrations (0.25 mol/L) disrupted the gel through excessive ionic shielding and dehydration. This study has provided insight into the individual and synergistic roles of Na^+^ and PPI in regulating AMP gelation, establishing a theoretical foundation for designing sodium-controlled, calcium-free pectin–protein gels. However, this study was limited to physicochemical and microstructural characterizations. Therefore, future studies should focus on the molecular dynamics of Na^+^-pectin interactions and the long-term stability of these gels, while potential applications include sodium-controlled, calcium-free gel systems for plant-based meat analogues and functional foods.

## Figures and Tables

**Table 1:** Texture characteristics of gels of AMP with different Na^+^ concentrations.

Na^+^ Concentration (mol/L)	Hardness (N)	Elasticity (mm)	Adhesiveness (N)	Chewiness (mg)	Cohesiveness
0.15	3.84 ± 0.05 ^e^		2.58 ± 0.01 ^d^		0.67 ± 0.01 ^a^
0.20	6.00 ± 0.15 ^d^		0.90 ± 0.04 ^e^		0.16 ± 0.01 ^e^
0.25	7.64 ± 0.17 ^b^	0.08 ± 0.01 ^c^	3.49 ± 0.11 ^b^	0.29 ± 0.01 ^b^	0.40 ± 0.00 ^c^
0.30	8.73 ± 0.13 ^a^	0.22 ± 0.01 ^a^	4.49 ± 0.01 ^a^	0.65 ± 0.02 ^a^	0.50 ± 0.01 ^b^
0.35	7.20 ± 0.03 ^c^	0.10 ± 0.01 ^b^	2.72 ± 0.01 ^c^	0.30 ± 0.03 ^b^	0.37 ± 0.01 ^d^

Note: Values are expressed as mean values (n = 3). Different lowercase letters within the same column indicate significant differences (*p* < 0.05).

**Table 2:** The hardness and WHC of AMP–PPI gels with different proportions.

AMP:PPI	Hardness (mm)	WHC (g Water/hg Gel)
0.1:7.5	4.0 ± 0.11 ^e^	42.35 ± 2.32 ^f^
0.2:7.5	6.18 ± 0.12 ^d^	52.96 ± 2.48 ^e^
0.3:7.5	7.26 ± 0.06 ^a^	66.32 ± 0.37 ^a^
0.4:7.5	6.83 ± 0.21 ^bc^	64.78 ± 1.55 ^ab^
0.5:7.5	6.81 ± 0.27 ^bc^	57.27 ± 1.4 ^cd^
0.1:10	5.64 ± 0.06 ^d^	53.61 ± 1.32 ^e^
0.2:10	7.06 ± 0.07 ^ab^	58.58 ± 1.17 ^cd^
0.3:10	7.02 ± 0.07 ^ab^	65.09 ± 0.96 ^a^
0.4:10	7.03 ± 0.06 ^b^	63.74 ± 0.8 ^ab^
0.5:10	6.86 ± 0.08 ^b^	59.05 ± 1.22 ^cd^
0.1:12.5	4.37 ± 0.04 ^e^	52.50 ± 0.55 ^e^
0.2:12.5	5.65 ± 0.05 ^d^	52.77 ± 0.7 ^e^
0.3:12.5	5.75 ± 0.06 ^cd^	58.15 ± 1.3 ^b^
0.4:12.5	5.87 ± 0.07 ^cd^	60.70 ± 1.03 ^cd^
0.5:12.5	5.65 ± 0.39 ^d^	58.95 ± 0.53 ^bc^

Note: Values are expressed as mean values (n = 3). Different lowercase letters within the same column indicate significant differences (*p* < 0.05).

**Table 3:** Texture characteristics of AMP–PPI composite gels with different Na^+^ concentrations.

Na^+^ Concentration (mol/L)	Hardness (N)	Elasticity (mm)	Adhesiveness (N)	Chewiness (mg)	Cohesiveness
0	7.23 ± 0.46 ^e^	0.12 ± 0.00 ^b^	2.43 ± 0.11 ^c^	0.31 ± 0.02 ^e^	0.26 ± 0.01 ^c^
0.05	10.43 ± 0.37 ^d^	0.13 ± 0.02 ^b^	3.06 ± 0.20 ^b^	0.52 ± 0.03 ^d^	0.29 ± 0.01 ^c^
0.10	13.80 ± 0.34 ^b^	0.17 ± 0.01 ^a^	3.53 ± 0.20 ^b^	0.79 ± 0.05 ^b^	0.33 ± 0.01 ^b^
0.15	15.56 ± 0.13 ^a^	0.18 ± 0.01 ^a^	5.20 ± 0.55 ^a^	0.88 ± 0.02 ^a^	0.41 ± 0.02 ^b^
0.20	12.40 ± 0.36 ^c^	0.12 ± 0.01 ^b^	3.34 ± 0.12 ^b^	0.60 ± 0.01 ^c^	0.32 ± 0.05 ^a^

Note: Values are expressed as mean values (n = 3). Different lowercase letters within the same column indicate significant differences (*p* < 0.05).

## Data Availability

The original contributions presented in the study are included in the article. Further inquiries can be directed to the corresponding authors.
